# Comparison of different thresholds of PSA density for risk stratification of PI-RADSv2.1 categories on prostate MRI

**DOI:** 10.1259/bjr.20210886

**Published:** 2021-11-09

**Authors:** Rossano Girometti, Gianluca Giannarini, Valeria Panebianco, Silvio Maresca, Lorenzo Cereser, Maria De Martino, Stefano Pizzolitto, Martina Pecoraro, Vincenzo Ficarra, Chiara Zuiani, Claudio Valotto

**Affiliations:** 1 Department of Medicine, Institute of Radiology, University of Udine, Santa Maria dellaMisericordia University Hospital, Udine, Italy; 2 Urology Unit, Santa Maria della Misericordia University Hospital, Udine, Italy; 3 Department of Radiological Sciences, Oncology and Pathology, Sapienza University of Rome, Rome, Italy; 4 Division of Medical Statistic, Department of Medicine, University of Udine, Udine, Italy; 5 Pathology Unit, Santa Maria della Misericordia University Hospital, Udine, Italy; 6 Department of Human and Paediatric Pathology “Gaetano Barresi”, Urologic Section,University of Messina, Messina, Italy

## Abstract

**Objectives:**

To compare the effect of different PSA density (PSAD) thresholds on the accuracy for clinically significant prostate cancer (csPCa) of the Prostate Imaging Reporting And Data System v.2.1 (PI-RADSv2.1).

**Methods:**

We retrospectively included 123 biopsy-naïve men who underwent multiparametric magnetic resonance imaging (mpMRI) and transperineal mpMRI-targeted and systematic prostate biopsy between April 2019 and October 2020. mpMRI, obtained on a 3.0T magnet with a PI-RADSv2.1-compliant protocol, was read by two radiologists (>1500/>500 mpMRI examinations). csPCa was defined as International Society of Urogenital Pathology grading group ≥2. Receiver operating characteristic analysis was used to calculate per-index lesion sensitivity, specificity, and area under the curve (AUC) of PI-RADSv.2.1 categories after adjusting for PSAD ≥0.10,≥0.15, and ≥0.20 ng/mL ml^−1^. Per-adjusted category cancer detection rate (CDR) was calculated, and decision analysis performed to compare PSAD-adjusted PI-RADSv.2.1 categories as a biopsy trigger.

**Results:**

csPCa prevalence was 43.9%. PSAD-adjustment increased the CDR of PI-RADSv2.1 category 4. Sensitivity/specificity/AUC were 92.6%/53.6%/0.82 for unadjusted PI-RADS, and 85.2%/72.4%/0.84, 62.9%/85.5%/0.83, and 92.4%/53.6%/0.82 when adjusting PI-RADS categories for a 0.10, 0.15, and 0.20 ng/ml ml^−1^ PSAD threshold, respectively. Triggering biopsy for PI-RADS four lesions and PSAD ≥0.10 ng/mL ml^−1^ was the strategy with greatest net benefit at 30 and 40% risk probability (0.307 and 0.271, respectively).

**Conclusions:**

PI-RADSv2.1 category four with PSAD ≥0.10 ng/mL ml^−1^ was the biopsy-triggering cut-off with the highest net benefit in the range of expected prevalence for csPCa.

**Advances in knowledge:**

0.10 ng/mL ml^−1^ is the PSAD threshold with higher clinical utility in stratifying the risk for prostate cancer of PI-RADSv.2.1 categories.

## Introduction

Over the last years, prostate magnetic resonance imaging (MRI) has been validated as a tool to minimize the detection of clinically indolent and maximize the detection of clinically significant, prostate cancer compared to conventional transrectal ultrasound-guided systematic biopsy.^1–5^ Moreover, the high sensitivity and low false-negative rate of prostate MRI account for a potential 30% reduction of unnecessary biopsies in men without clinically significant prostate cancer (csPCa).^6^ There is intense research on risk-adapted strategies for the proper selection of candidate to prostate biopsy and the reduction of false-negative results.^7^

On the other hand, unnecessary biopsy may result from MRI lesions that are associated with a low detection rate of csPCa, such as the Prostate Imaging–Reporting And Data System (PI-RADS) category 3 findings, which harbor csPCa in 25% of cases only.^8^ Most successful attempts to stratify csPCa risk inherent to category 3 observations have relied on PSA density (PSAD), which has been advocated as a tool to differentiate between PI-RADS 3 lesions requiring biopsy (high PSAD, need to avoid false-negatives) or not (low PSAD, need to avoid false-positives).^9^ Adjusting PI-RADS categories for PSAD also showed the potential to increase cancer detection rate (CDR) in PI-RADS 1–2 categories,^3,10,11^ suggesting that PSAD-based adjustments might be of value in different PI-RADS categories.

PI-RADS 4 category is an additional source of false-positives, as suggested by the relatively low CDR found in a study by Tan et al^12^ (54%) and a systematic review by Barkovich et al^13^ (48%). Little is known on the effect of PSAD adjustment of category 4 lesions, as they have been usually merged with category 5 ones in previous studies.^14^ Moreover, while 0.15 ng/ml ml^−1^ is the most commonly used PSAD cut-off in the radiological literature, different PSAD thresholds and ranges have been proposed to stratify individual patient risk. After pooling the results of five studies on biopsy-naïve patients, Schoots et al^6^ recently evaluated the effect of adjusting PI-RADS categories with different PSAD thresholds (0.10, 0.15, and 0.20 ng/ml ml^−1^), showing a progressive increase in false-negatives and decrease in false-positives as the cut-off increased. To our knowledge, only one previous multicenter study by Falagario et al^15^ compared those thresholds in the same population of roughly 2500 men. On decision analysis, the authors found that performing biopsy for highly suspicious imaging findings (PI-RADS or Likert 4–5 categories), and/or suspicious imaging findings (PI-RADS or Likert 3 category) combined with PSAD > 0.20 ng/mL ml^−1^ was the strategy achieving the highest net benefit in biopsy-naïve subjects. However, study results have been obtained using mixed interpretation criteria, and in the focused scenario of the Prostate MRI Outcome Database. As far as we know, no previous studies compared PSAD thresholds in the context of more homogeneous and standardized interpretation criteria.

The purpose of our study was to investigate the clinical utility of different thresholds of PSAD in improving the accuracy of PI-RADS categories for detecting csPCa on mpMRI and mpMRI-targeted and systematic biopsy.

## Methods and materials

### Patients

This study was approved by the Institutional Review Board of the Department of Medicine, University of Udine. A waiver for informed consent acquisition was obtained in view of the retrospective design. By setting April 2019 to October 2020 as the reference period, we performed a computerized search to identify all men who underwent mpMRI at our institution because of persistently increased serum PSA level (≥3 ng ml^−1^ over at least two repeated samples) and/or suspicious digital rectal examination. In all patients, serum PSA level was obtained ≤1 month before mpMRI. Of 355 resulting subjects, we included 166 biopsy-naïve men who underwent prostate biopsy at our institution ≤3 months from mpMRI because of PI-RADS ≥3 observations, or family history of csPCa regardless of a negative mpMRI result (PI-RADS 1–2). Of note, PSAD was not used to stratify PI-RADS categories in clinical practice. PI-RADS categories were attributed using v.2.1 (PI-RADSv.2.1).

Exclusion criteria were absence of contrast administration due to contraindications (*n* = 7), poor image quality due to artifacts from air in the rectum (*n* = 2), and ongoing therapy with 5-alfa reductase inhibitor at the time of mpMRI (*n* = 21). The latter exclusion criterion was adopted to prevent potential effects of the therapy on diffusion-weighted imaging (DWI) (*e.g.* reduced conspicuity on DWI images)^16,17^ and avoid the confounder of calculating PSAD over a prostate gland volume potentially reduced by the effect of therapy.^18^ We also excluded 13 men in whom mpMRI was not interpreted by the reference readers. The final cohort, thus, included 123 men.

### mpMRI protocol

Examinations were performed on a 3.0T magnet (Achieva, Philips) with a 32-channel surface coil after rectal enema and intramuscular administration of 20 mg hyoscine butylbromide as an antispasmodic agent (except when contraindicated). Sequences and acquisition parameters are shown in Supplementary Material 1. Using the vendor software, the apparent diffusion coefficient (ADC) map was built by fitting signal intensity versus all the b-values of the DWI sequence with a maximum b-value of 1000 s/mm^2^. Dynamic contrast-enhanced (DCE) imaging was performed after intravenous administration of 0.1 mmol/Kg gadoteridol (Prohance, Bracco) at an injection rate of 3 ml s^−1^, using a remote-controlled power injector (Medrad Spectris Solaris EP). Subtraction images were automatically provided for the analysis (pre-contrast acquisition subtracted to each post-contrast acquisition).

Supplementary Material 1.

### Image analysis

Analysis was performed using the mpMRI reports provided during clinical activity by one of two readers, that is, reader 1 (R1) and reader (R2) with an overall mpMRI experience of >1500 examinations and >500 examinations in total, respectively. Both R1 and R2 have been serving as reference radiologists in the prostate multidisciplinary group of our institution (one meeting per week). Readers evaluated mpMRI images on a picture and archive communication system (PACS) workstation (SuiteEstensa, Esaote), using PI-RADSv.2.1 criteria for categorizing image findings and calculating prostate volume (Supplementary Material 1).^19^ In our institutional practice, mpMRI lesions are marked on the PI-RADSv.2.1 sector map attached to the report, as well directly as on axial *T*_2_-weighted and mpMRI images. Given the clinical scenario in which examinations were performed, R1 and R2 were unblinded to indication to mpMRI and clinical information, including PSAD. An independent radiologist with 3 years of experience in prostate imaging (<500 examinations in total) collected all the mpMRI reports and re-assessed the images on the same PACS station in order to identify the index lesion for each examination, defined as the lesion showing the highest PI-RADSv.2.1 category in the original report. In the case of ≥2 lesions with the same category, the largest one was assumed to represent the index lesion. The examination was categorized as PI-RADSv.2.1 1 when no lesions were reported. Index lesions were assumed to be suspicious for csPCa when categorized as PI-RADSv.2.1 ≥3.

### Prostate biopsy and standard of reference

Prostate biopsy was performed under local anesthesia via transperineal approach by a single urologist with 2 years of experience using the Aplio 300 platform (Toshiba/Canon) with rigid image registration and electromagnetic needle tracking. Targeted biopsy of PI-RADS ≥3 lesions was performed first, deploying a total of 4 cores (two in-target and two peri-target). Subsequent systematic biopsy included the conventional 12-core template. Histological analysis of biopsy samples was the standard of reference. One of three genitourinary pathologists (5–30 years of experience) performed analysis according to International Society of Urological Pathology (ISUP) criteria.^20^ csPCa was defined as ISUP grading group ≥2 on targeted and/or systematic biopsy.

### Statistical analysis

Variables were reported with descriptive statistics, using median and interquartile range (IQR) values and percent proportions with 95% confidence intervals (95% CI). We calculated the CDR of targeted biopsy, defined as the per-patient prevalence of csPCa over PI-RADS ≥3 index lesions, and of targeted plus systematic biopsy, defined as the per-patient prevalence of csPCa in targeted cores and/or systematic cores. CDR was stratified by PI-RADSv2.1 category ≤2, 3, 4, and 5, as well as for three different thresholds of PSAD, namely, ≥0.10,≥0.15, and ≥0.20 ng/ml ml^−1^.

By matching the results of mpMRI and biopsy according to the rules shown in Supplementary Material 1, we run receiver operating characteristic (ROC) analysis to assess per-patient sensitivity, specificity, and area under the curve (AUC) of unadjusted and PSAD-adjusted PI-RADSv2.1 categories for csPCa, as shown in Supplementary Material 1. For instance, PI-RADSv2.1 category 3 was divided into 3l (low PSAD) and 3h (high PSAD), depending on whether PSAD exceeded, or not, the established cut-off (*e.g.* 0.10 ng/ml ml^−1^). PI-RADSv.2.1 and PI-RADSv.2.1-adjusted category achieving the highest Youden index was set as the cut-off for maximizing both sensitivity and specificity.^21^ AUCs obtained at different PSAD thresholds were compared using the DeLong method. Positive-predictive value (PPV) and negative-predictive value (NPV) were calculated as well. Alfa level for statistical significance was set at 0.05.

Finally, we used the decision analysis^22^ to quantify the clinical utility of different models potentially determining the need for targeted plus systematic biopsy in our series, that is*,* biopsy for no patients (“treat none” strategy), biopsy for all patients (“treat all” strategy), and biopsy for index observations with the following categories: PI-RADSv.2.1 category 3, category 3h, category 4, and category 4h. The clinical utility of each model was measured as the net benefit at 10%, 20%, 30%, and 40% risk probability of csPCa, and was plotted over the 0–99% range of risk probability in order to build decision analysis curves. The net benefit expresses the percent gain in true-positives adjusted for false-positive results, given a certain threshold probability of harboring csPCa.^23,24^ The decision analysis was repeated for each PSAD reference level.

All analyses were performed with commercially available software (MedCalc Software v.19.8 Ltd, Stata Statistical Software, v.17). Decision analysis was run on Stata using source codes freely available at https://www.mskcc.org/departments/epidemiology-biostatistics/biostatistics/decision-curve-analysis. The standard of reference for ROC analysis and decision analysis was the combination of targeted plus systematic biopsy.

## Results

Median patient age, serum PSA level and PSAD value were 67.0 years (IQR 67.0–72.5), 6.16 ng ml^−1^ (IQR 6.1–9.3) and 0.12 ng/mL ml^−1^ (IQR 0.12–0.17), respectively.

R1 and R2 evaluated 82/123 (66.7%) and 41/123 (33.3%) examinations, respectively. Index lesions were categorized as PI-RADSv.2.1 category 1 in 18 cases (14.6%), category 2 in 12 cases (9.7%), category 3 in 11 cases (8.9%), category 4 in 47 cases (38.2%), and category 5 in 35 cases (28.4%). Of the 123 men, those with category ≥3 index lesions (75.6%) underwent targeted biopsy plus systematic biopsy, while the remaining 30 men with negative mpMRI underwent systematic biopsy only. csPCa was found in 54/123 men (43.9%; 95% CI 40.5–70.4), with 27/54 ISUP 2 cancers (50.0%), 17/54 ISUP 3 cancers (31.5%), 6/54 ISUP 4 cancers (11.1%), and 4/54 ISUP 5 cancers (7.4%). Thirteen other men (10.5%; 95% CI 6.9–22.2) were diagnosed with ISUP 1 cancers. Of men referred to biopsy, 14 also showed 14 non-index suspicious lesions [category 3 in four cases (28.5%), and category 4 in 10 cases (71.5%)], all of which underwent additional targeted biopsy with a final diagnosis of csPCa in 10/14 cases (71.4%). Positivity of the non-index lesions was associated with true-positive index lesions in all cases. Details on CDR are shown in Table 1. Nine out of 47 category 4 assignments were upgraded from the initial category 3. Three out of nine cases were true-positive ISUP 2 cancers with PSAD ≥0.10 ng/mL ml^−1^, while the remaining six cases were false-positives (PSAD ≤0.10 and>0.10 ng/mL ml^−1^ in 3 and 3 cases, respectively).

**Table 1.** Cancer detection rate for clinically significant prostate cancer on a per Prostate Imaging – Reporting And Data System v.2.1 basis with and without stratification by PSA density

PI-RADSv2.1 category of index lesion (N.patients)	PSAD threshold (ng/ml ml^−1^)	PSAD value (ng/ml ml^−1^) (N.patients)	CDR for csPCa on targeted biopsy (%)	CDR for csPCa on target +systematic biopsy (%)	Number of false-positives on targeted biopsy (%)
≤2 (30)	0.10	Any (30)	NA	2/30 (6.7)	NA
		<0.10 (16/30)	NA	1/16 (6.2)	NA
		≥0.10 (14/30)	NA	1/14 (7.1)	NA
	0.15	Any (30)	NA	2/30 (6.7)	NA
		<0.15 (26/30)	NA	1/26 (3.8)	NA
		≥0.15 (4/30)	NA	1/4 (25.0)	NA
	0.20	Any (30)	NA	2/30 (6.7)	NA
		<0.20 (27/20)	NA	2/27 (7.4)	NA
		≥0.20 (3/30)	NA	0/3 (0.0)	NA
3 (11)	0.10	Any (11)	2/11 (18.1)	2/11 (18.2)	9/11 (81.8)
		<0.10 (6/11)	2/6 (33.3)	2/6 (33.3)	4/6 (66.7)
		≥0.10 (5/11)	0/5 (0.0)	0/5 (0.0)	5/5(100%)
	0.15	Any (11)	2/11 (18.2)	2/11 (18.2)	9/11 (81.8)
		<0.15 (10/11)	2/10 (20.0)	2/10 (20.0)	8/10 (80.0)
		≥0.15 (1/11)	0/1 (0.0)	0/1 (0.0)	1/1 (100.0)
	0.20	Any (11)	2/11 (18.2)	2/11 (18.2)	9/11 (81.8)
		<0.20 (10/11)	2/10 (20.0)	2/10 (20.0)	8/10 (80.0)
		≥0.20 (1/11)	0/1 (0.0)	0/1 (0.0)	1/1 (100.0)
4 (47)	0.10	Any (47)	20/47 (42.5)	21/47 (44.7)	27/47 (57.5)
		<0.10 (17/47)	4/17 (23.5)	4/17 (23.5)	13/17 (76.5)
		≥0.10 (30/47)	16/30 (53.3)	17/30 (56.7)	14/30 (46.7)
	0.15	Any (47)	20/47 (42.5)	21/47 (44.6)	27/47 (57.5)
		<0.15 (38/47)	15/38 (39.4)	16/38 (42.1)	23/38 (60.6)
		≥0.15 (9/47)	5/9 (55.5)	5/9 (55.5)	4/9 (44.5)
	0.20	Any (47)	20/47 (42.5)	21/47 (44.6)	27/47 (57.5)
		<0.20 (43/47)	19/43 (44.1)	20/43 (46.5)	24/43 (55.9)
		≥0.20 (4/47)	1/4 (25.0)	1/4 (25.0)	3/4 (75.0)
5 (35)	0.10	Any (35)	29/35 (82.8)	29/35 (82.8)	6/35 (17.2)
		<0.10 (5/35)	3/5 (60.0)	3/5 (60.0)	2/5 (40.0)
		≥0.10 (30/35)	26/30 (86.6)	26/30 (86.6)	4/30 (13.4)
	0.15	Any (35)	29/35 (82.8)	29/35 (82.8)	6/35 (17.2)
		<0.15 (10/35)	8/10 (80.0)	8/10 (80.0)	2/10 (20.0)
		≥0.15 (25/35)	21/25 (84.0)	21/25 (84.0)	4/25 (16.0)
	0.20	Any (35)	29/35 (82.8)	29/35 (82.8)	6/35 (17.2)
		<0.20 (15/35)	11/15 (73.3)	11/15 (73.3)	4/15 (26.7)
		≥0.20 (20/35)	18/20 (90.0)	18/20 (90.0)	2/20 (10.0)

CDR, Cancer detection rate; NA, Not applicable; PI-RADSv2.1, Prostate imaging–reporting and data system version 2.1; PSAD, PSA density; csPCa, Clinically significant prostate cancer.

### Diagnostic accuracy of PI-RADSv2.1 categories adjusted or not for PSAD

**Figure 1.**
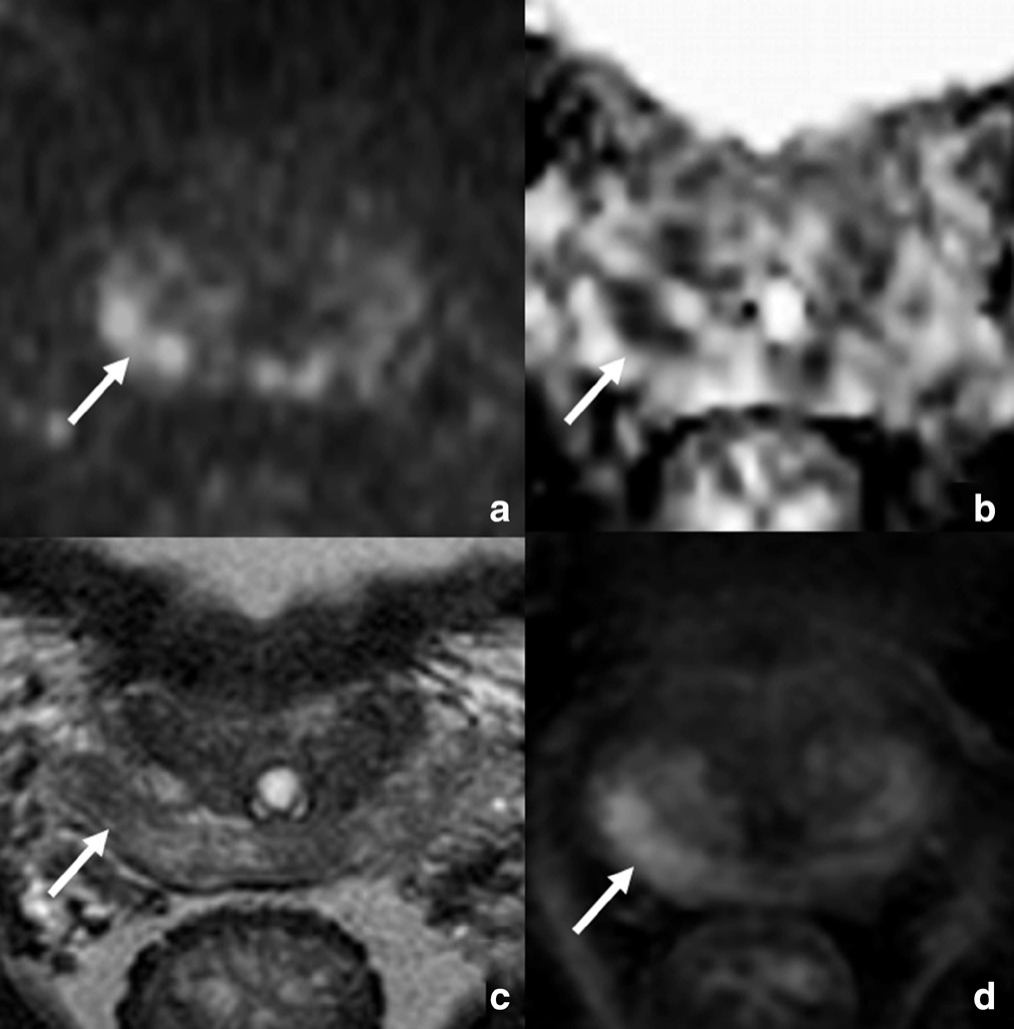
True-positive PI-RADS 4 index lesion with high PSAD (0.33 ng/ml ml^−1^) in a 61-year-old male with an International Society of Urogenital Pathology (ISUP) grading group 2 cancer on targeted biopsy. On multiparametric magnetic resonance imaging (mpMRI), the index lesion appeared as a 12 mm focal zone of restricted diffusion in the lateral posterior peripheral zone of the right base, with hyperintensity on the *b* = 2000 sec/mm^2^ image (arrow in a), and hypointensity on the apparent diffusion coefficient (ADC) map (arrow in b). This finding also showed moderate hypointensity on corresponding axial T2-weighted image (arrow in c), and focal enhancement on subtracted dynamic contrast-enhanced imaging (arrow in d).

**Figure 2.**
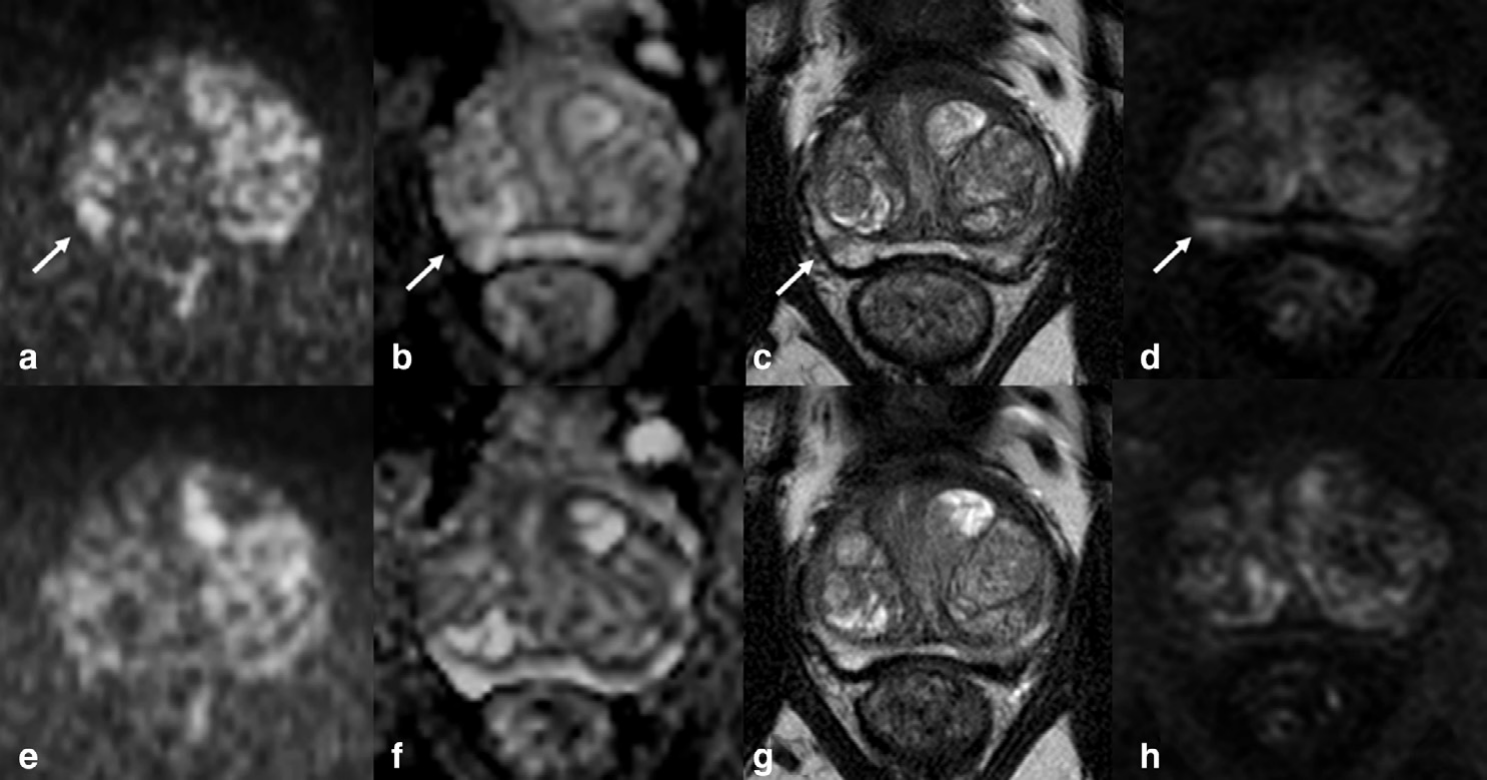
False-positive PI-RADS four index lesion in a 64-year-old patient with low PSAD (0.09) ng/ml ml^−1^ and negative-targeted and systematic biopsy. mpMRI showed a 5 mm focus of restricted diffusion in the lateral posterior peripheral zone of the right mid-gland, appearing as an hyperintense focus on high b-value image (arrow in a), and tiny focal hypointensity on the ADC map (arrow in b) corresponding to mild hypointensity on axial T2-weighted imaging (arrow in c) and focal contrast-enhancement on dynamic contrast-enhanced imaging (arrow in d). Of note, the lesion was no longer visible on the same sequences (e-h) of a subsequent mpMRI performed 6 months after the biopsy. On this basis, the lesion was retrospectively assessed as focal prostatitis.

On ROC analysis, PI-RADSv2.1 categorization achieved 92.6% sensitivity (95% CI 82.1–97.7%) and 53.6% specificity (95% CI 41.2–65.7%) for a category ≥3 threshold, corresponding to an AUC of 0.82 (95%CI 0.74–0.88). Sensitivity, specificity, and AUC for PSAD-adjusted categorization were 85.2% (95%CI 72.9–93.4%), 72.5% (95%CI 60.4–82.5%), and 0.84 (95%CI 0.77–0.90) at ≥0.10 ng/ml ml^−1^, and 62.9% (95%CI 48.7–75.7%), 85.5% (95%CI 75.0–92.8%), and 0.83 (95%CI 0.75–0.89) at ≥0.15 ng/ml ml^−1^ (Supplementary Figure 2). At both PSAD levels, the Youden index corresponded to a PI-RADSv2.1 ≥4h cut-off. In the case of ≥0.20 ng/ml ml^−1^ threshold, 92.6% sensitivity (95% CI 81.8–97.9%), 53.6% specificity (95% CI 41.2–65.7%), and 0.82 AUC (95% CI 0.74–0.88%) were achieved for a PI-RADSv2.1 ≥4l cut-off. Sensitivity and specificity obtained at each PSAD threshold are reported in Supplementary Table 4. Figures 1 and 2 illustrate example cases.

Pairwise comparison between AUCs showed significant difference between PSAD ≥0.10 ng/ml ml^−1^- versus ≥0.20 ng/ml ml^−1^-adjusted categorization (*p* = 0.041). Other comparisons were not significant (*p* = 0.115 for PSAD values ≥ 0.10 versus ≥0.15 ng/ml ml^−1^, and *p* = 0.376 for ≥0.15 versus ≥0.20 ng/ml ml^−1^).

### Decision analysis

**Figure 3.**
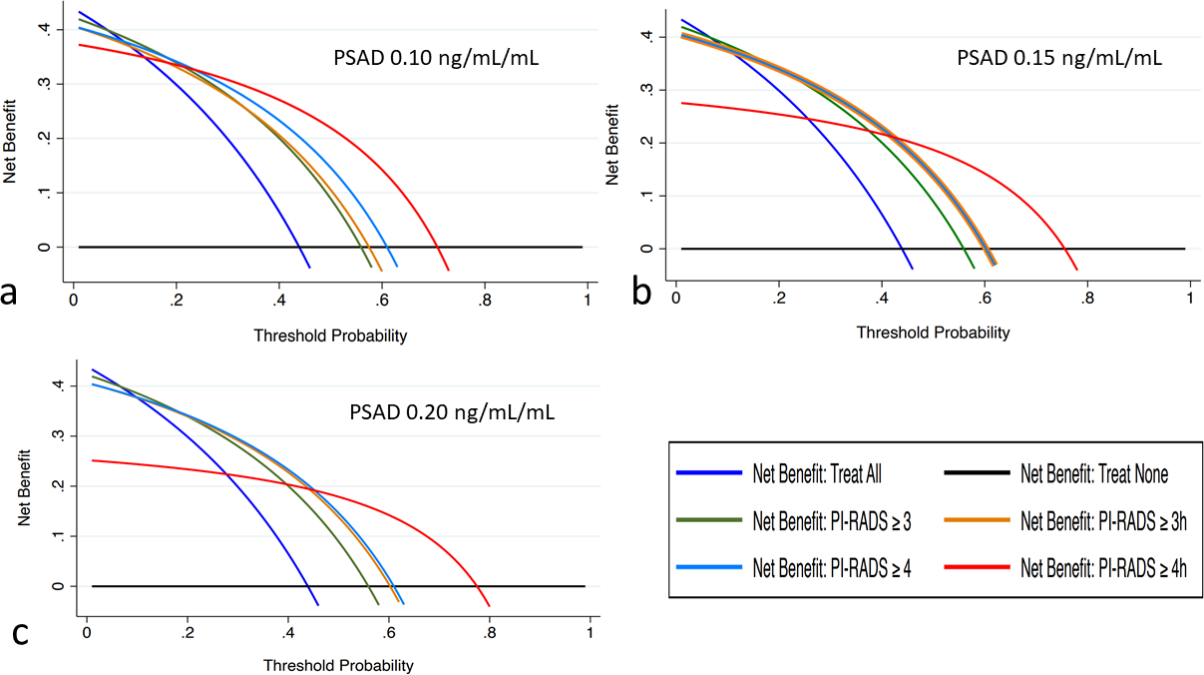
Decision analysis curves for the biopsy models corresponding to different PSAD thresholds, namely, ≥0.10 ng/ml ml^−1^ (**a**),≥0.15 ng/ml ml^−1^ (**b**), and ≥0.20 ng/ml ml^−1^ (**c**). 3 = unadjusted PI-RADS 3 category; 3*h* = PI RADS 3 category with high PSAD; 4 = PI RADS 4 category; 4*h* = PI RADS 4 category with high PSAD.

When comparing the net benefit of different PI-RADS thresholds triggering biopsy, we obtained decision analysis curves as shown in Figure 3. Absolute net benefit values at different PSAD-adjusted levels are reported in Table 2. PI-RADS 4h category adjusted for PSAD ≥0.10 ng/ml ml^−1^ was the biopsy trigger with the greatest net benefit for risk probabilities corresponding to the expected prevalence of csPCa in unselected biopsy-naïve patients. Table 3 provides an overview of false-positive and false-negative cases using unadjusted and PSAD-adjusted PI-RADSv.2.1 categories. Regardless of the PSAD threshold used, most false-negatives were ISUP 2 cancers.

**Table 2.** Decision analysis showing the expected benefit of performing biopsy using the reported Prostate Imaging – Reporting And Data System v.2.1 categories for 10–40% threshold probabilities

PSAD threshold (ng/ml ml^−1^)	Risk probability	Net benefit according to the PI-RADSv2.1 cutoff				
		3	3h	4	4h	Treat all
0.10	10%	0.385	0.373	0.377	0.356	0.376
	20%	0.339	0.331	0.341	0.335	0.298
	30%	0.279	0.277	0.295	0.307	0.198
	40%	0.200	0.206	0.233	0.271	0.065
0.15	10%	0.385	0.376	0.376	0.266	0.376
	20%	0.339	0.339	0.339	0.254	0.298
	30%	0.279	0.291	0.291	0.238	0.198
	40%	0.200	0.227	0.227	0.216	0.065
0.20	10%	0.385	0.376	0.377	0.243	0.376
	20%	0.339	0.339	0.341	0.233	0.298
	30%	0.279	0.291	0.295	0.220	0.198
	40%	0.200	0.227	0.233	0.203	0.065

PI-RADSv2.1, Prostate imaging – reporting and data system version 2.1; PSAD, PSA density; 3*h*, PI-RADSv2.1 category three with PSAD above the established threshold; 4*h*, PI-RADSv2.1 category four with PSAD above the established threshold.

“Treat all” is assumed to represent the strategy of performing prostate biopsy to all patients.

**Table 3.** PSA density-adjusted and non-adjusted per-patient number of clinically significant prostate cancers missed for each Prostate Imaging–Reporting And Data System v.2.1 category potentially auctioning prostate biopsy

PSAD (ng/ml ml^−1^)	PI-RADSv2.1 cut-off	PPV% (95% CI)	NPV% (95% CI)	TP patients (N)	TN patients (N)	FP patients (N)	FN patients (N)				
							ISUP ≥**2**	ISUP 2	ISUP 3	ISUP 4	ISUP 5
0.10	3	55.9 (41.7–73.3)	93.3 (62.0–100)	52	28	41	2	0	2	0	0
	3h	58.1 (43.1–76.6)	89.2 (61.4–100)	50	33	36	4	2	2	0	0
	4	61.0 (45.2–80.4)	90.2 (63.5–100)	50	37	32	4	2	2	0	0
	4h	70.8 (51-8–94.4)	86.2 (63.9–100)	46	50	19	8	4	4	0	0
0.15	3	55.9 (41.7–73.3)	93.3 (62.0–100)	52	28	41	2	0	2	0	0
	3h	60.2 (44.7–79.4)	90.0 (63.0–100)	50	36	33	4	2	2	0	0
	4	61.0 (45.2–80.4)	90.2 (63.5–100)	50	37	32	4	2	2	0	0
	4h	77.3 (53.5–100)	74.7 (56.8–96.3)	34	59	10	20	12	6	2	0
0.20	3	55.9 (41.7–73.3)	93.3 (62.0–100)	52	28	41	2	0	2	0	0
	3h	60.2 (44.7–79.4)	90.0 (63.0–100)	50	36	33	4	2	2	0	0
	4	61.0 (45.2–80.4)	90.2 (63.5–100)	50	37	32	4	2	2	0	0
	4h	76.9 (51.9–100)	71.4 (54.5–91.9)	30	60	9	24	15	6	3	0

FN, False negative; FP, False positive; ISUP, International Society of Urogenital Pathology; NPV, Negative predictive value; PI-RADSv2.1, Prostate imaging – reporting and data system version 2.1; PPV, Positive predictive value; PSAD, PSA density; TN, True negative; TP, True positive; 3h, PI-RADSv2.1 category three with PSAD above the established threshold; 4h, PI-RADSv2.1 category four with PSAD above the established threshold.

International Society of Urogenital Pathology grading group are provided for false-negative cancers.

## Discussion

We observed that the adjustment of PI-RADSv.2.1 categories for a PSAD threshold ≥0.10 ng/ml ml^−1^ achieved higher AUC for csPCa than other values (≥0.15 and≥0.20 ng/ml ml^−1^) and unadjusted PI-RADSv2.1 categorization. The latter showed unbalanced high sensitivity and low specificity, in line with a recent meta-analysis on PI-RADSv2.1 accuracy (94 and 56% pooled sensitivity and specificity for a category ≥3 cut-off, respectively).^25^ Combining category 4h and PSAD ≥0.10 ng/ml ml^−1^ as a trigger for biopsy was the strategy with the highest net benefit on decision analysis when the probability of csPCa was assumed to be 30 and 40%, that is, within the expected range of csPCa prevalence (28–49%, average 39%) in biopsy-naïve patients.^6^ This was not the case for strategies adjusting for PSAD of 0.15 and 0.20 ng/ml ml^−1^, for which category 3h or four showed comparable net benefit.

Similarly to a study by Falagario et al^15^ with a comparable csPCa prevalence (40.19%) and median PSAD (0.13 ng/ml ml^−1^), we confirmed the added value of PSAD in stratifying csPCa risk. However, in that study, the authors found greater clinical utility of different strategies with or without PSA adjustment, or with different PSAD thresholds, namely biopsying PI-RADSv2.1/Likert category ≥4 lesions and/or PI-RADSv2.1/Likert category ≥3 lesions if PSAD >0.20 ng/ml ml^−1^ (main strategy), or PI-RADSv2.1/Likert ≥ 4 findings and/or PI-RADSv2.1/Likert ≥ 4 findings if PSAD >0.10 and/or >0.20 ng/ml ml^−1^ (secondary strategy). While obtained on a small study population, our findings suggest a more definite approach combining more uniform interpretation criteria (PI-RADSv2.1) with a lower PSAD threshold.

In our series, the adjustment for PSAD reduced false-positive assignments, as witnessed by increased PPV of adjusted PI-RADSv2.1 categories potentially triggering biopsy (3h and 4h) compared to unadjusted categories (3 and 4). CDR increased, too, with a higher prevalence of cancers in category ≥4 assignments associated with high PSAD than those with low PSAD, regardless of the PSAD threshold used. While the overall trend for category 4 and 5 lesions as a whole is in line with previous reports,^2,6,26–29^ the stratified analysis showed that the gain from PSAD-adjustment was low for category 5, as the per-category CDR increased only slightly, that is, from 82.8% to 84.0–90.0% depending on the PSAD threshold used. This was an expected result, given the high risk for csPCa inherent to PI-RADSv2.1 5 lesions, for which CDR has been reported 72–73% in previous studies.^13,30^

On the contrary, the CDR for the unadjusted category four was relatively low (42.5%). This value compares to the CDR shown in systematic reviews by Barkovich et al^13^ (48.0%) and Schoots^30^ (39.0% for ISUP ≥2 cancers), suggesting that PI-RADS 4 assignments are a frequent false-positive source of unnecessary biopsy. While we were not able to identify clinical and mpMRI-related features associated with false-positive category 4 assignments, we showed that category 4 was the one benefiting the most from PSAD-adjustment, with CDR increasing from 42.5–44.6% to 55.5–56.6% when using ≥0.10–0.15 ng/ml ml^−1^ thresholds. This effect was not found for a PSAD ≥0.20 ng/ml ml^−1^, possibly because only a few men with category 4 index lesions showed a PSA level above that threshold (*n* = 4). Of note, PSAD-adjustment did not affect the CDR of category 3 lesions. This might be explained by the low number of category 3 assignments in our series, accounting for 11/123 (8.9%) index observations. As PI-RADS three lesions are expected to be comparably low (<10%) in a high volume center,^9^ our results corroborate the concept that in this ideal scenario the main target to minimize false-positives might be PI-RADS 4 category.

After pooling different studies using PSAD to stratify PI-RADS categories, Schoots et al^6^ showed that 31% of men with high-risk mpMRI findings (PI-RADS 4–5 observations) are expected to harbor csPCa despite low PSAD (<0.10 ng/mL ml^−1^). This would be the base for recommending biopsy in categories 4–5 regardless of PSAD. Our results are difficult to compare, as we did not merge the two categories. Using a PSAD ≥0.10 ng/mL ml^−1^ and PI-RADS 4h as a potential cut-off for triggering biopsy, we showed an NPV (86.2%) approaching the value expected for category 3 as the trigger for biopsy (90.8%).^31^ This combination corresponded to a lower proportion of false-negative csPCa cases compared to stratifying category 4 for higher PSAD values, and to a 70.7% PPV, which is significantly higher than the 40% pooled PPV recently reported in the meta-analysis by Mazzone et al^32^. Our results should be interpreted with caution, given the need for validation in larger prospective series. However, we believe they might represent a basis for further research, especially regarding how the risk inherent to each PI-RADS-adjusted category may impact on the decision to biopsy.

We acknowledge some limitations to our study. First, our study population is relatively small and has been collected on a monocentric basis. Further prospective studies on larger series should be performed to validate our findings. Second, the retrospective use of clinical mpMRI readings for the analysis implied unblinding to PSAD. One might argue that the knowledge of PSAD might have influenced mpMRI readings, for example by inducing the radiologist to consider a finding as more focal and/or conspicuous on DWI in the case of higher PSAD. We believe that similar potential effects on image analysis were reasonably limited by the fact that PSAD has no impact on the PI-RADS decision rules.^19^ However, our results might be of limited generalizability in a setting in which image analysis is performed with the Likert assessment, which can be influenced by prior knowledge of PSAD.^33^ Second, we estimated the prostate gland volume for calculating PSAD according to the PI-RADSv2.1 rules.^19^ Using volumetric segmentation as the standard of reference, Ghafoor et al^34^ found that our method slightly overestimated the prostate volume compared to the PI-RADSv2.0 method.^35^ While differences were minimal in absolute terms, the PI-RADSv2.0 method was advocated as a more reliable tool to calculate PSAD. Another study on 397 patients^36^ showed that the PI-RADS v2.1-derived volume did not affect the accuracy of PSAD for ISUP ≥2 cancers compared to the segmentation-derived volume, although the accuracy for ISUP ≥4 cancers was greater when PSAD was calculated with the latter method. Overall, although our findings are reasonably generalizable in a PI-RADS v.2.1-centered context, they should be confirmed in a setting using segmentation-derived volumes as the surrogate standard of reference.^37^ Third, our results were obtained on a per-patient rather than per-lesion basis, thus possibly overemphasizing the sensitivity and PPV. On the other hand, we observed 14 suspicious non-index lesions, all of which underwent additional targeted biopsy with a final diagnosis of csPCa in most cases. This suggests that sensitivity inflation, if any, would be reasonably limited. Finally, we used full mpMRI, thus making the results not generalizable to the setting of biparametric MRI, for which *ad hoc* studies should be performed.

## Conclusions

Our study showed that PSAD ≥0.10 ng/ml ml^−1^ was the threshold with the greatest clinical utility in stratifying PI-RADSv.2.1 categories for csPCa. This threshold corresponded to higher AUC and better balance between sensitivity and specificity than PSAD ≥0.15 or ≥0.20 ng/ml ml^−1^. On decision analysis, PI-RADSv2.1 category 4 adjusted for PSAD ≥0.10 ng/mL ml^−1^ was the biopsy trigger with the greatest net benefit for risk probabilities corresponding to the expected prevalence of csPCa in unselected biopsy-naïve men. If confirmed in larger prospective studies, our results might emphasize the concept that the combination of lower PSAD threshold and higher PI-RADSv2.1 cut-off may allow better risk stratification for referral to prostate biopsy.
