# Supplementary material for: Comparison of different thresholds of PSA density for risk stratification of PI-RADSv2.1 categories on prostate MRI
Source: Br J Radiol. 2021 Nov 9;95(1131):20210886. doi: 10.1259/bjr.20210886 (PMC8978227; doi:10.1259/bjr.20210886)
Supplement: Supplementary Material 1. [file bjr.20210886.suppl-01.docx]

**SUPPLEMENTARY MATERIAL**


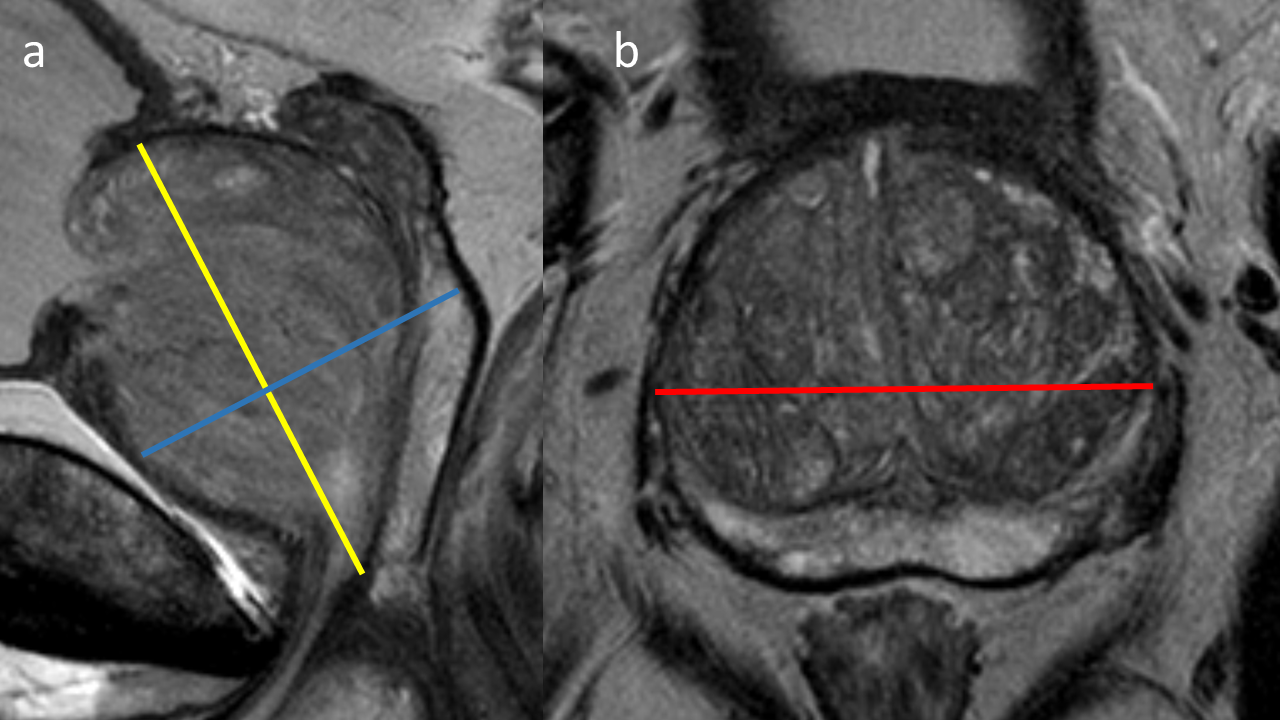


Supplementary Figure 1. Criteria for calculating prostate volume, and in turn the prostate antigen level density (PSAD), using a midsagittal T2-weighted image (a) and axial T2-weighted image, respectively. The gland volume was estimated in mL with the ellipsoid formula, i.e. by multiplying maximum anteroposterior diameter (blue line in a) x maximum longitudinal diameter (yellow line in a) x maximum transverse diameter (red line in b) x 0.52.


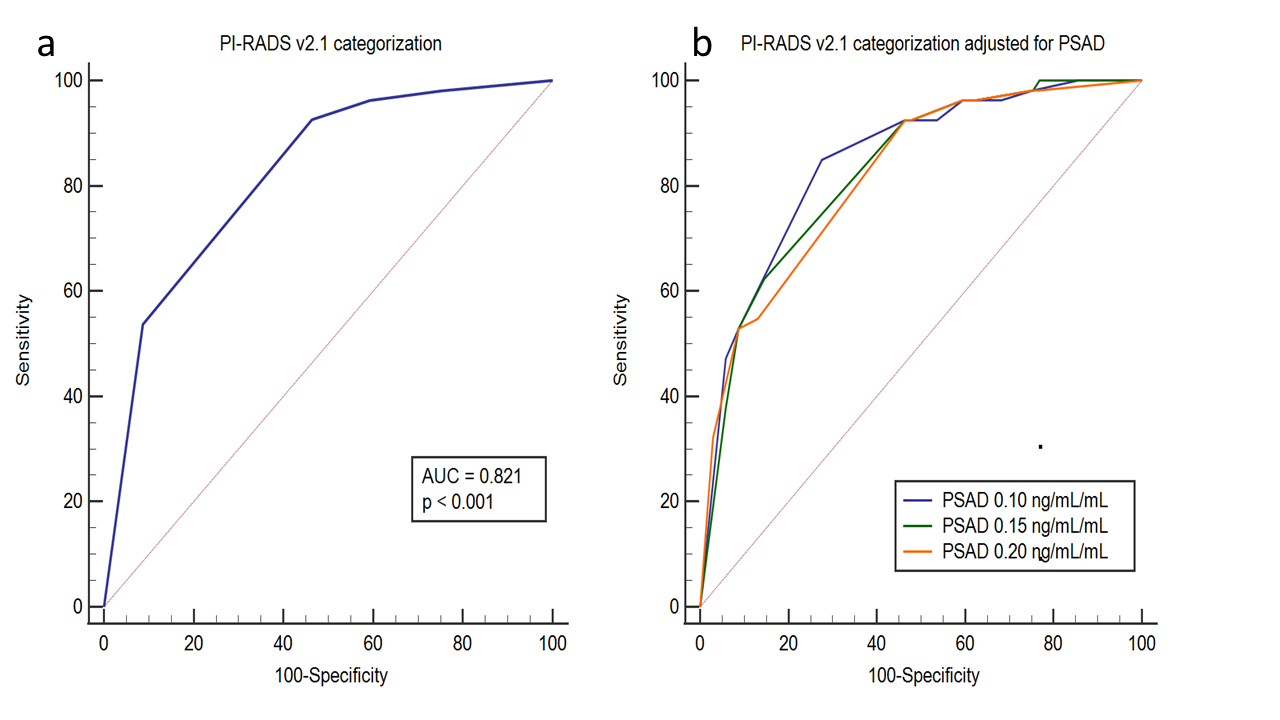


Supplementary Figure 2. Receiver operating characteristics (ROC) analysis plots for unadjusted Prostate imaging reporting and data system version 2.1 (PI-RADSv2.1) categorization of imaging findings (a), as well as PI-RADSv2.1 categorization after adjusting for the reported prostate antigen level density (PSAD) thresholds.

|  | **DWI double sequence** | | **TSE-T2WI** | **DCE** |
| --- | --- | --- | --- | --- |
|  | **First**  **sequence*** | **Second sequence**** |  |  |
| **Sequence** | SS-EPI | SS-EPI | TSE | THRIVE |
| **Weighting** | DWI | DWI | T2 | T1 |
| **Acquisition plane** | Transverse | Transverse | Transverse/ coronal/sagittal | Transverse |
| **TR (ms)** | 5350 | 5424 | 4727/3076/3714 | 3.5 |
| **TE (ms)** | 68 | 78 | 80/80/80 | 1.77 |
| **Echo train length** | - | - | 8/16/16 | - |
| **EPI factor** | 109 | 109 | - | - |
| **Half scan factor** | 0.62 | 0.62 | No | no |
| **FOV (mm x mm)** | 200 x 200 | 200 x 200 | 180x180/180x180/180x180 | 200x200 |
| **Acquisition voxel size** | 2 x 2 x 3 | 2 x 2 x 3 | 0.6 x 0.6 x 3 | 1.2 x 1.2 x 8 |
| **Reconstruction pixel size (mm x mm x mm)** | 1.4 x 1.4 x 3 | 1.4 x 1.4 x 3 | 0.45 x 0.45 x 3 | 0.63 x 0.63 x 4 |
| **Number of slices** | 24 | 24 | 24/20/20 | 20 |
| **Interslice gap (mm)** | 0 | 0 | 0/0/0 | 0 |
| **b-values**  **(s/mm^2^)/number of excitations** | 100/1, 500/1, 1000/2 | 100/1, 2000/3 | - | - |
| **Number of excitations** | - | - | 2/1/1 | 1 |
| **Fat saturation** | SPAIR | SPAIR | - | Spectral fat saturation |
| **Parallel imaging**  **(x acceleration factor)** | SENSE x 2 | SENSE x 2 | SENSE x 2.5/1/1 | SENSE x 2 |
| **Acquisition time (min)** | 3.2 | 3.2 | 5.4/4.3/5.1 | 6.16 |

Supplementary Table 1. Acquisition parameters of multiparametric prostate magnetic resonance imaging. The total acquisition time (6.16 min) reported for dynamic contrast-enhanced imaging (DCE) resulted from 44 serial acquisitions of a T1-weighted high-resolution isotropic volume (THRIVE) sequence with 8.4 s acquisition time each.

DWI = diffusion-weighted imaging; TSE-T2WI = turbo spin echo T2-weighted imaging; SS-EPI = single-shot echo-planar imaging; TR = time of repetition; EPI = echo-planar imaging; TE = time of echo; FOV = field of view; SENSE = sensitivity encoding; SPAIR = spectral adiabatic inversion recovery; * used to calculate the apparent diffusion coefficient map; ** used to provide b = 2000 s/mm^2^ images.

| **Category of the index lesion at mpMRI** | **Results of**  **targeted biopsy** | **Results of**  **systematic biopsy** | **Per-patient categorization** |
| --- | --- | --- | --- |
| PI-RADS v2.1 ≤ 2 | Not applicable | Positive for csPCa | False negative |
|  | Not applicable | Negative for csPCa | True negative |
| PI-RADS v2.1 ≥ 3 | Positive for csPCa | Positive or negative  for csPCa | True positive |
|  | Negative for csPCa | Positive or negative  for csPCa | False positive |

Supplementary Table 2. Categorization of multiparametric magnetic resonance imaging findings for the purpose of calculating sensitivity, specificity, positive predictive value, and negative predictive value.

mpMRI = multiparametric magnetic resonance imaging; PI-RADS v2.1 = Prostate Imaging – Reporting And Data System version 2.1; csPCa = clinically significant prostate cancer.

| **PI-RADS v2.1 category for index**  **lesion** | **PSAD cut-off**  **(ng/ml ml^-1^)** | | |
| --- | --- | --- | --- |
|  | **≥0.10** | **≥0.15** | **≥0.20** |
| 1 | 1l | 1l | 1l |
|  | 1h | 1h | 1h |
| 2 | 2l | 2l | 2l |
|  | 2h | 2h | 2h |
| 3 | 3l | 3l | 3l |
|  | 3h | 3h | 3h |
| 4 | 4l | 4l | 4l |
|  | 4h | 4h | 4h |
| 5 | 5l | 5l | 5l |
|  | 5h | 5h | 5h |

Supplementary Table 3. Overview of the subcategories obtained by adjusting Prostate Imaging – Reporting And Data System version 2.1 categories at each PSA density threshold. Resulting subcategories were entered in receiver operating characteristics analysis, thus obtaining three curves for each PSA density.

PI-RADS v2.1 = Prostate Imaging – Reporting And Data System version 2.1; PSAD = PSA density; l = low PSA density (below the stablished cut-off); h = high PSA density (equal to or above the established cut-off).

|  | **PSAD threshold**  **(ng/ml ml^-1^)** | | | | | |
| --- | --- | --- | --- | --- | --- | --- |
| **PSAD-adjusted PI-RADS v2.1 category** | **≥0.10** | | **≥0.15** | | **≥0.20** | |
|  | **Sensitivity %**  **(95%CI)** | **Specificity %**  **(95%CI)** | **Sensitivity %**  **(95%CI)** | **Specificity %**  **(95%CI)** | **Sensitivity %**  **(95%CI)** | **Specificity %**  **(95%CI)** |
| >1l | 100.0  (93.4-100.0) | 14.5  (7.2-25.0) | 100.0  (93.4-100.0) | 23.2  (13.9-34.9) | 98.2  (89.9-100.0) | 23.2  (13.9-34.9) |
| >1h | 98.2  (90.1-100.0) | 24.6  (15.1-36.5) | 98.2  (90.1-100.0) | 24.6  (15.1-36.5) | 98.2  (89.9-100.0) | 24.6  (15.1-36.5) |
| >2l | 96.3  (87.3-99.5) | 31.9  (21.2-44.2) | 96.3  (87.3-99.5) | 37.7  (26.3-50.2) | 96.3  (87.0-99.5) | 37.7  (26.3-50.2) |
| >2h | 96.3  (87.3-99.5) | 40.6  (28.9-53.1) | 96.3  (87.3-99.5) | 40.6  (28.9-53.1) | 96.3  (87.0-99.5) | 40.6  (28.9-53.1) |
| >3l | 92.6  (82.1-97.9) | 46.4  (34.3-58.8) | 92.6  (82.1-97.9) | 52.2  (39.8-64.4) | 92.6  (81.8-97.9) | 52.2  (39.8-64.4) |
| >3h | 92.6  (82.1-97.9) | 53.6  (41.2-65.7) | 92.6  (82.1-97.9) | 53.6  (41.2-65.7) | 92.6  (81.8-97.9) | 53.6  (41.2-65.7) |
| >4l | 85.2  (72.9-93.4) | 72.5  (60.4-82.5) | 62.9  (48.7-75.7) | 85.5  (75.0-92.8) | 54.7  (40.4-68.4) | 86.9  (76.7-93.9) |
| >4h | 53.7  (39.6-67.4) | 91.3  (82.0-96.7) | 53.7  (39.6-67.4) | 91.3  (82.0-96.7) | 52.8  (38.6-66.7) | 91.3  (82.0-96.7) |
| >5l | 48.2  (34.3-62.2) | 94.2  (85.8-98.4) | 38.9  (25.9-53.1) | 94.2  (85.8-98.4) | 32.1  (19.9-46.3) | 97.1  (89.9-99.6) |
| ≥5h | 0.0  (0.0-6.6) | 100.0  (94.8-100.0) | 0.0  (0.0-6.6) | 100.0  (94.8-100.0) | 0.0  (0.0-6.7) | 100.0  (94.8-100.0) |

Supplementary Table 4. Overview of sensitivity and specificity for clinically significant prostate cancer achieved by using PSAD-adjusted PI-RADS v2.1 categories at receiver operating characteristics analysis.

PI-RADS v2.1 = Prostate Imaging – Reporting And Data System version 2.1; PSAD = PSA density.
